# COP1 regulates the stability of CAM7 to promote photomorphogenic growth

**DOI:** 10.1002/pld3.144

**Published:** 2019-06-25

**Authors:** Dhirodatta Senapati, Ritu Kushwaha, Siddhartha Dutta, Jay Prakash Maurya, Srabasthi Biswas, Sreeramaiah N. Gangappa, Sudip Chattopadhyay

**Affiliations:** ^1^ Department of Biotechnology National Institute of Technology Durgapur India

**Keywords:** Arabidopsis, CAM7 and COP1, gene expression, photomorphogenesis, protein stability

## Abstract

The unique member of the calmodulin gene family, Calmodulin7 (CAM7), plays a crucial role as transcriptional regulator to promote Arabidopsis seedling development. CAM7 regulates the expression of *HY5*, which is intimately involved in the promotion of photomorphogenic growth and light‐regulated gene expression. COP1 ubiquitin ligase suppresses photomorphogenesis by degrading multiple photomorphogenesis promoting factors including HY5 in darkness. Genetic interaction studies, in this report, reveal that *CAM7* and *COP1* co‐ordinately work to promote photomorphogenic growth and light‐regulated gene expression at lower intensity of light. CAM7 physically interacts with COP1 in the nucleus. Further, in vivo study suggests that CAM7 and COP1 interaction is light intensity dependent. We have also shown that functional COP1 is required for optimum accumulation of CAM7 at lower fluences of light. Taken together, this study demonstrates the coordinated function of CAM7 and COP1 in Arabidopsis seedling development.

## 1 INTRODUCTION

Light controls multiple developmental processes in plant life cycle (Deng & Quail, 1999; Franklin & Quail, 2010; Jiao, Lau, & Deng, 2007; Kami, Lorrain, Hornitschek, & Fankhauser, 2010; Wang & Deng, 2003). Following germination, seedlings grown in the dark displaying skotomorphogenic growth, which is characterized by long hypocotyl, closed cotyledon, and apical hook; while those in the light, exhibit photomorphogenic growth characterized by short hypocotyl with open and expanded cotyledons (Arsovski, Galstyan, Guseman, & Nemhauser, 2012; Chen, Chory, & Fankhauser, 2004). Several positive and negative regulators have been functionally characterized that work downstream to multiple photoreceptors and are intimately involved in the regulation of Arabidopsis seedling development (Briggs & Olney, 2001; Brown et al., 2005; Chen & Chory, 2011; Heijde & Ulm, 2012; Jiao et al., 2007; Kleine, Lockhar, & Batschauer, 2003; Lin, 2002; Neff, Fankhauser, & Chory, 2000; Quail, 2002). The functional connectivity among a fairly large number of regulatory proteins started to be unravelled to understand the complex light signaling network.

Calmodulin (CaM) is a small and highly conserved ubiquitous protein. CaM contains two helix‐loop‐helix EF‐hand in each of its two globular domains (Chin & Means, 2000; Klee & Vanaman, 1982; Yamniuk & Vogel, 2004). In contrast to animal cells, which have a single isoform of *CaM* encoded by three separate genes (Fischer et al., 1988), plants contain multiple *CaM* genes encoding several CaM isoforms with a few amino acid differences (Cho et al., 1998; Choi et al., 2002; Lee, 2005; Mc Cormack, Tsai, & Braam, 2005). In Arabidopsis, seven genes encode four CAM isoforms, of which CAM1/CAM4 differ by four amino acid substitutions from CAM7, whereas CAM2/3/5 and CAM6 differ by one amino acid position as compared to CAM7 (Mc Cormack et al., 2005).

Among these seven bona fide calmodulin proteins in Arabidopsis, CAM7/ZBF3 specifically binds to the Z‐ and G‐box of light‐regulated promoters (Kushwaha, Singh, & Chattopadhyay, 2008). CAM7 acts as a positive regulator of photomorphogenesis under a wide spectrum of light such as red, far‐red, and blue light (Kushwaha et al., 2008). ELONGATED HYPOCOTYL5 (HY5), a constitutively nuclear localized bZIP transcription factor, has also been shown to function as a positive regulator of photomorphogenesis under various wavelengths of light, including red, far‐red, and blue light, and more recently in UV‐B light as well (Binkert et al., 2014; Chattopadhyay, Puente, Deng, & Wei, 1998; Osterlund, Hardtke, Wei, & Deng, 2000; Oyama, Shimura, & Okada, 1997; Ulm et al., 2004). The *hy5* mutant seedlings show partially etiolated phenotype at various wavelengths of light (Ang & Deng, 1994; Ang et al., 1998; Koornneef, Rolff, & Spruit, 1980). Although *cam7* mutants do not display altered photomorphogenic growth, *cam7 hy5* double mutants display a super‐tall phenotype at various wavelengths of light (Kushwaha et al., 2008). Recent studies have shown that CAM7 and HY5 physically interact with each other and bind to the E‐ and T/G‐box of *HY5* promoter, respectively, to promote photomorphogenic growth (Abbas, Maurya, Senapati, Gangappa, & Chattopadhyay, 2014).

The CONSTITUTIVELY PHOTOMORPHOGENIC (COP)/DEETIOLATED/FUSCA proteins are repressors of photomorphogenesis (Jiao et al., 2007; Lau & Deng, 2012; Wei & Deng, 1999). The *cop1* mutant seedlings show photomorphogenic growth in the dark and develop a less number of lateral roots as compared to wild‐type plants (Deng, Caspar, & Quail, 1991; Deng & Quail, 1999). COP1 acts as an E3 ubiquitin ligase and targets photomorphogenesis‐promoting factors such as HY5, HYH, LAF1, HFR1, BIT1, and BBX22 for degradation in the dark (Chang, Maloof, & Wu, 2011; Holm, Ma, Qu, & Deng, 2002; Osterlund et al., 2000; Saijo et al., 2003; Seo et al., 2003; Yang, Lin, Hoecker, et al., 2005; Yang, Lin, Sullivan, et al., 2005). However, GBF1/ZBF2, a bZIP transcription factor of blue light signaling, is degraded in the dark by a proteasomal pathway independent of COP1 and SPA1 (Mallappa, Singh, Ram, & Chattopadhyay, 2008). Furthermore, COP1 is required to maintain the higher level of accumulation of GBF1 in light (Mallappa et al., 2008; Maurya, Sethi, Gangappa, Gupta, & Chattopadhyay, 2015; Singh, Ram, Abbas, & Chattopadhyay, 2012). Besides showing its activity in the dark, COP1 also degrades several photoreceptors in the light (Jang, Henriques, Seo, Nagatani, & Chua, 2010; Seo, Watanabe, Tokutomi, Nagatani, & Chua, 2004). In contrast with its functions under red, far red, and blue light, COP1 acts as a positive regulator of HY5 in UV‐B light‐induced photomorphogenesis (Binkert et al., 2014; Heijde & Ulm, 2012; Oravecz et al., 2006). COP1 is more abundant in the nucleus in the dark, however, it migrates to cytosol upon light exposure, which results in the accumulation of target proteins to promote photomorphogenesis (Osterlund & Deng, 1998; Pacín, Legris, & Casal, 2013; Subramanian et al., 2004; Von Arnim & Deng, 1994). A COP1 suppressor, CSU1, has recently been shown to play a major role in maintaining the COP1 homeostasis in the dark (Xu et al., 2014).

In this study, we have investigated the genetic and biochemical interactions between CAM7 and COP1. We have also analyzed the stability of CAM7 mediated by COP1 during Arabidopsis seedling development. Our data strongly suggest that CAM7 and COP1 genetically and physically interact with each other and work in a cooperative manner. While their genetic interactions show an additive role of CAM7 and COP1, molecularly CAM7 is stabilized by COP1 at a lower intensity of light for promotion of photomorphogenesis.

## 2 METHODS

### 2.1 Plant material, growth conditions and generation of double mutants

The wild‐type *Arabidopsis thaliana*,* cam7* mutant and *cop1‐4*and *cop1‐6* used in this study are in the Col‐0 background. *CAM7‐3MycOE*, transgenic lines were generated as described by Kushwaha et al. (2008). *Arabidopsis thaliana* seeds were surface‐sterilized with 2% sodium hypochlorite and 0.05% triton‐X solution, sown on MS plates, kept at 4°C in darkness for 3 to 5 days, and transferred to specific light conditions at 22°C.

The *cam7 cop1* double mutant was constructed by genetic crosses, using *cop1‐6* or *cop1‐4* allele and *cam7‐1* single mutant. In the F2 generation, plants with *cop1* mutant phenotype were selected, which confirmed the mutation for *cop1* locus, whereas for *cam7* mutation, PCR using gene‐specific primer LP15 and RP15 was utilized. F3 seedlings were further confirmed by genomic‐ and RT‐PCR and designated as corresponding double mutants.

For the generation of *CAM7 promoters–GUS* transgenic lines 1.1 Kb upstream to start codon was PCR amplified using primers FP and RP and was cloned in pBI101.2 between restriction sites. Orientation of the construct was confirmed by restriction digestion, and DNA sequence was confirmed by sequencing. This promoter–GUS construct was introduced into *Agrobacterium* strain *GV3101* and finally into Arabidopsis WT plants by vacuum infiltration. A homozygous line was generated and was further used for study.

Transgenic seedling overexpressing CAM7 in *cop1* mutant was generated by genetic crosses using *cop1* single mutant as female and *CAM7OE* transgenic lines as male in each of the individual crosses. Seedlings with *cop1* mutant phenotype were selected in F2 populations and the overexpression of *CAM7‐cMyc* transgene in *cop1* mutant was confirmed by western blot (using anti‐cMyc antibodies). Several homozygous lines were reconfirmed in F3 generation and were used for further studies.

### 2.2 Nuclear localization studies

Subcellular localization was performed as described in Von Arnim and Deng (1994). The 425 bp cDNA of CAM7 was amplified by PCR using primers and with NcoI and SpeI restriction sites at ends and cloned into *pCAMBIA1303‐GUS*. This construct was introduced into WT *Arabidopsis* plant by vacuum infiltration method. Homozygous lines expressing *CAM7‐GUS* were produced by selection on hygromycin and staining. Hypocotyl cells of six‐day seedling were visualized using Fluorescent Microscope (Nikon EFD3). The location of β‐glucuronidase activity was determined, using X‐gluc and the nuclei were identified using the DNA‐specific stain DAPI (Hoechest stain; 1 μg/ml).

### 2.3 Chlorophyll and anthocyanin measurements

Chlorophyll and anthocyanin levels were measured following protocols as described by Holm et al. (2002). Briefly, seedlings were collected into microcentrifuge tubes, weighed, and crushed by a pestle in 700 ml of chilled 80% acetone. Cellular debris was removed by centrifugation at 4°C, and the supernatant containing chlorophyll was collected into a fresh microcentrifuge tube, and volume was made up to 1 ml. Then the absorbance was measured at the wavelengths of 645 and 663 nm. The total chlorophyll content was calculated with the following formula: Chl A = 12.7 (A663) – 2.69 (A645), Chl B = 22.9 (A645) – 4.48 (A663).

About 20–30 seedlings were taken into a microcentrifuge tube, weighed; 400 μl of extraction solution (1% HCL in Methanol) was added and kept at Cold–dark condition. Next day, the seedlings were crushed, 200 μl of sterile water and 200 μl of chloroform were added. The debris was removed by centrifugation and supernatant was collected into a fresh microcentrifuge tube. Then spectrophotometric estimation was carried out by taking readings at the wavelengths of 530 nm and 657 nm. The total Anthocyanin content was calculated with the help of the following formula: (A_530_‐0.33A_657_)/gm of tissue.

### 2.4 Real‐time PCR

Wild‐type, mutant and transgenic seedlings were grown in constant dark and white light for 6 days. The total RNA was extracted using the RNeasy plant mini kit (Qiagen). However, cDNA was synthesized from 1 μg of total RNA using RT‐AMV reverse transcriptase (Thermo‐ Scientific). Real‐time PCR analyses were performed using the Thermal Cycler Applied Biosystem Step One and Light Cycler Fast start DNA Master plus SYBRGreen 1 systems (Applied Biosystem). The fold expression of different genes was determined using gene‐specific primers. The common 2^−ΔΔ*CT*^ algorithm was used to analyze the relative changes in gene expression. *ACTIN2*, a housekeeping gene was used as the endogenous control. Δ*Ct* value is calculated by normalizing samples Ct to Actin2 *Ct* values. This value for different samples was then normalized to *Ct* value of the experimental control (such as wild‐type and the ΔΔ*Ct* value was obtained. Fold expression was calculated by the formula, 2^−ΔΔ*CT*^, which was plotted on the graph.

### 2.5 In vitro‐binding assay

Full length coding sequence of *HY5* and *CAM7* proteins were cloned into *pGEX‐4T2* vector to yield a fusion with the Glutathione S‐transferase (GST) protein. These GST‐CAM7 and GST‐HY5 (which was used for the positive control) were overexpressed in *E. coli BL21 (DE3)* cells and purified by Glutathione sepharose 4B beads (Amersham Biosciences). Full‐length *COP1 CDS* was cloned into pET‐20b (+) vector with 6× Histidine tag at the C‐terminal of the protein. This full length COP1‐His protein was overexpressed in *E. coli* BL21 (DE3) cells and purified by Ni‐NTA Agarose beads (Qiagen). For in vitro‐binding assays, 2 μg of COP1‐HIS was individually bound to Ni‐NTA beads by incubating with in vitro pull down buffer (50 mM Tris‐Cl PH 7.5, 100 mM NaCl, 0.2% glycerol, 0.1% triton‐×100, 1 mM EDTA, 1 mM PMSF, 0.1% NP‐40 and 1× protease inhibitors cocktail (Sigma)) for 2 hr at 4°C. Excess unbound proteins were washed off and GST‐HY5, GST‐CAM7, GST protein was added in an equimolar ratio and incubated in 300 μl in vitro‐binding buffer at 4°C for overnight. The beads and supernatant were collected separately by brief centrifugation, and beads were washed three times with 1 ml of pull down buffer. The pellet was resuspended in 5× SDS loading buffer, boiled for 10 min, and analyzed by SDS‐PAGE. Both pellet and supernatant (2%) were analyzed by probing with anti‐GST antibodies (Sigma).

### 2.6 Yeast‐two hybrid assay

To generate constructs for yeast two‐hybrid assays, *CDS of HY5* (encoding full length protein) and CAM7 (encoding full length and truncated proteins) were cloned into pGADT7 vector to produce translational fusions with the activation domain. Similarly, the *CDS of COP1* (encoding full length protein) was cloned into *pGBKT7* vector (Clonetech Laboratories, Inc.,) to produce translational fusion with DNA‐binding domain. The constructs were transformed into yeast strain AH109 according to the Clonetech protocol. The protein‐protein interactions were examined by β‐galactosidase assays using CPRG (chlorophenol red‐β‐d‐galactopyranoside) as a substrate. The relative β‐galactosidase activities were calculated according to Clontech instructions. Expression of AD‐CAM, AD‐HY5 and BD‐COP1 fusion protein was examined by probing with the anti‐HA and anti c‐MYC antibodies, respectively.

### 2.7 BiFC assays

For BiFC experiments, the full‐length coding sequence of *CAM7* was cloned in the pUC‐SPYCE vector to produce a fusion protein of CAM7‐YFPC‐ter and the full‐length coding sequence of *COP1* was cloned in the *pUC‐SPYNE* vector to obtain the COP1‐YFPN‐ter fusion. The desired constructs were mixed in equal proportion (5 μg each) and co‐bombarded into onion epidermal cells using the helium 465 driven particle accelerator (PDS‐1000) following the manufacturer's instructions (Bio‐Rad) as described in Abbas et al. (2014). The bombarded onion peels were kept in the dark for approximately 20 hr at 22°C to allow the expression of the transfected DNA and reconstruction of the functional YFP, and then mounted onto glass slide and observed under confocal laser469scanning microscope (Leica‐TCS‐SP‐2) with visible AOTF (Aquistic optical tunablefilter) standard filter. Empty BiFC vectors (*pSPYCE‐35S* + *pSPYNE‐35S* or cYFP + nYFP) and their combinations with CAM7‐cYFP (CAM7‐cYFP + nYFP) and COP1‐nYFP (COP1‐nYFP + cYFP) were co‐transformed into onion cells as negative control. 4, 6‐Diamidino‐2‐phenylindole staining was performed to identify the nuclei.

### 2.8 Co‐immunoprecipitation assays

Total protein from wild‐type and *CAM7*‐overexpresser (with c‐Myc tag) lines was extracted from seedlings grown in constant darkness for 6 days or grown in different intensities of WL (15 and 100 μmol m^−2 ^s^−1^), in a buffer containing (400 mM sucrose, 50 mM Tris‐Cl, pH 7.5,10% glycerol, 2.5 mM EDTA). 500 μg of total protein of each line was used for co‐immunoprecipitation in co‐immunoprecipitation buffer (50 mM Tris‐Cl, 100 mM NaCl, 10% glycerol, 5 mM EDTA, 0.1% Triton X‐100, 0.2% Nonidet P‐40) using 10–15 μl of anti‐c‐Myc polyclonal antibody(Sigma) for 6 hr at 4°C. Then 30 μl of preblocked protein A‐agarose beads (Sigma) were added and further incubated for 2 hr at 4°C. After the beads were washed three times with co‐immunoprecipitation buffer, they were kept in a boiling water bath in 5× protein loading dye for 10 min and then run in SDS‐PAGE. Both pellet and supernatant (5%) were analyzed by probing with anti‐COP1 antibody. Immunoblot analysis of plant protein with anti‐c‐Myc antibody (Sigma) was performed to show the prey protein.

### 2.9 Proteasomal assays

For this experiment, 5‐day‐old dark‐grown *CAM7OE* seedlings were treated with MG132 or mock treated with 0.1% DMSO for 12 hr. The seedlings were then washed and incubated at various intensities of WL. Total protein was extracted and subjected to immunoblot analysis.

### 2.10 Western blot analysis

After separating the protein samples on SDS‐PAGE gel, they were transferred to Nitrocellulose membrane (Amersham protran) at 130 mA for 1 hr in transfer buffer (Tris48 mM, Glycine 39 mM, 20% methanol pH 9.2) in trans‐blot semi‐Dry (Amersham Biosciences) module. The membrane was stained with Ponceau‐S to confirm the protein transfer and then washed with sterile MQ water. The membrane was then incubated for 1 hr in 25 ml blocking buffer (5% nonfat dry milk in PBS and 0.05% Tween‐20) at room temperature on a rotary shaker. The blocking reagent was removed and the affinity Purified primary antibody diluted (1:250 to 1:10,000) in 10 ml PBS with 0.05% Tween‐20 was added and incubated for 2 hr with shaking at room temperature. The membrane was then washed thrice with 25 ml of wash buffer (PBS and 0.05% Tween‐20) for 5 min each. The secondary antibody conjugated with HRP diluted (1:5,000 to 10,000) in 10 ml PBS with 0.05% Tween‐20, was added and incubated for 1 hr with shaking at room temperature. The membrane was washed thrice with 25 ml of wash buffer each time at room temperature. Western blot was performed using the Super signal west Pico chemiluminescent substrate kit (Pierce) and following the instructions as provided by the manufacturer. Substrate working solution was prepared by mixing peroxide solution and Luminol/enhancer solution in 1:1 ratio and the blot was incubated in that working solution for 5 min in the dark. The blot was then removed from the working solution and covered with saran wrap in cassette and exposed to X‐ray film for different times depending on signal strength.

### 2.11 Primers used in various experiments

List of primers used in in vitro pull down assays

CAM7‐FL: FP (BamHI): CGGGATCCATGGCGGATCAGCTAACCGATGACCCAG
CAM7‐FL: RP (XhO1): CCGCTCGAGCTTTTGGTTCAATAAATTACTTTTCTCAG
HY5‐FL: FP (NdeI): GGAATTCCATATGCAGGAACAAGCGACTAG CTCTTT
HY5‐FL: RP (ClaI): CCATCGATTCAAAGGCTTGCATCAGCATTAG
COP1‐FL: FP (EcoRI): GCGAATTCATGGAAGAGATTTCGACGGATC
COP1‐FL: RP (PstI): CG*GGATCC*TCACGCAGCGAGTACCAGAAC

List of primers used in yeast two hybrid assays

CAM7‐FL FP (NdeI) GGAATTCCATATGGCGGATCAGCTAACCGATGAC
CAM7‐FL RP (BamHI) CGGGATCCTCACTTTGCCATCATGACTTTGACG
HY5‐FL FP (EcoRI) GGAATTCATGCAGGAACAAGCGACTAGC
HY5‐FL RP (BamHI) CGGGATCCTCAAAGGCTTGCATCAGC
COP1‐FL FP (EcoRI) GGAATTCATGGAAGAGATTTCGACGG
COP1‐FL RP (PstI) AACTGCAGAGCTCGGTATAAATCTATTC

List of primers used in BiFC assays

CAM7‐FL: FP (BamHI) CGGGATCCATGGCGGATCAGCTAACCGATGACCAG
CAM7‐FL: RP (XhO1) CCGCTCGAGCTTTGCCATCATGACTTTGACG
COP1‐ FL: FP (Asc1) GGCGCGCCATGGAAGAGATTTCGACGGATC
COP1‐FL: RP (Xho1) CCGCTCGAGCGCAGCGAGTACCAGAACTTTG

List of primers used in Real time PCR

CAB(FP): CCCATTTCTTGGCTTACAACAAC
CAB(RP): TCGGGGTCAGCTGAAAGTCCG
RBCS(FP): GAGTCACACAAAGAGTAAAGAAG
RBCS(RP): CTTAGCCAATTCGGAATCGGT
CHS(FP) –ATGGTGATGGCTGGTGCTTC
CHS(RP)‐TTAGAGAGGAACGCTGTGCAAG
CAM7_iFP: TTTGACAAGGACCAGAACGG
CAM7cmyc_RP2: CAAGTCTTCCTCGGAGATTAG

## 3 RESULTS

### 3.1 Higher level of CAM7 in *cop1* mutant background enhances the photomorphogenic growth in the dark and at various wavelengths of light

**Figure 1:**
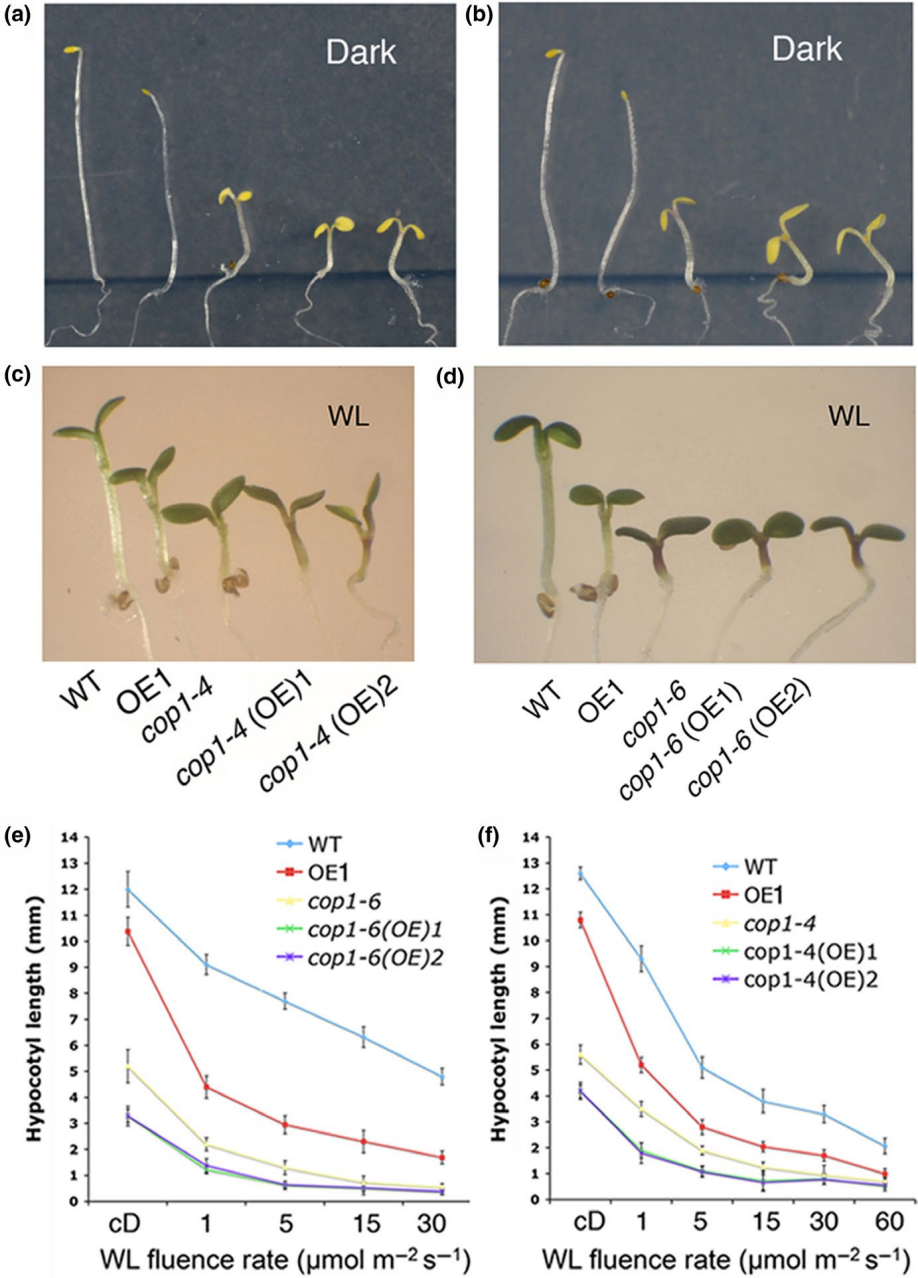
CAM7 overexpression in *cop1* enhances the hyperphotomorphogenic growth of *cop1* in dark and white light. (a, b) Visible phenotypes of 6‐day‐old wild‐type, mutants and transgenic seedlings grown in constant dark. (c, d) Visible phenotypes of 6‐day‐old wild‐type, mutants and transgenic seedlings grown in constant WL (15 μmol m^−2^ s^−1^). *CAM7OE cop1‐6* and *CAM7OE cop1‐4* are indicated as *cop1‐6(OE)* and *cop1‐4(OE),* respectively. (e, f) Quantification of hypocotyl length of 6‐day‐old seedlings grown in constant dark or at various fluences of WL. Around 25–30 seedlings were taken for the measurement of hypocotyl length. The error bars indicate standard deviation. The experiments were repeated for at least three times with consistent results and a representative result is shown

It has been shown earlier that overexpression of CAM7 leads to partial photomorphogenic growth in the dark, and displays hyper‐photomorphogenic growth at various wavelengths of light (Kushwaha et al., 2008). Since *cop1* mutants display photomorphogenic growth in the dark and hypersensitivity to light (Ang & Deng, 1994), we ask whether CAM7 is functionally connected to COP1. To address this question, we introduced *CAM7‐cMyc* transgene from overexpresser transgenic lines (in wild‐type background; Kushwaha et al., 2008) into *cop1‐4* and *cop1‐6* allelic mutants individually by genetic crosses (Ang & Deng, 1994). We examined the hypocotyl length of 6‐day‐old *cop1 CAM7OE* transgenic seedlings in the dark and at various fluences of white light (WL). The increased accumulation of CAM7 in *cop1‐4* and *cop1‐6* mutants (*cop1‐4 CAM7OE* and *cop1‐6 CAM7OE*) exhibited shorter hypocotyl than *CAM7OE* transgenic lines, and *cop1‐4* and *cop1‐6* mutants in the dark or WL (Figure 1a–d). Quantification of the hypocotyl length revealed that the shorter hypocotyl phenotype is more prominent at lower fluences of WL (Figure 1e,f). To determine the effect of CAM7 accumulation on *cop1* mutant phenotype at specific wavelength of light, 6‐day‐old seedlings were grown at various wavelengths of light such as red light (RL), far red light (FR), and blue light (BL). The *cop1‐4 CAM7OE* and *cop1‐6 CAM7OE* transgenic lines displayed enhanced inhibition of hypocotyl elongation compared to *cop1‐4* and *cop1‐6* mutants at various wavelength of light tested (Figure S1). Taken together, these results suggest that higher level of CAM7 in *cop1* enhances the photomorphogenic growth of *cop1* mutants in the dark and at various wavelengths of light. In order to determine the level of *CAM7* expression at various transgenic backgrounds tested in this study, we carried out qRTPCR. As shown in Figure S2, the level of expression of *CAM7* was found to be similar in various backgrounds.

Although *cam7* mutant does not exhibit any phenotype, *cam7 hy5* double mutants displayed super tall phenotype at various wavelengths of light indicating a synergistic function of CAM7 and HY5 (Kushwaha et al., 2008). We therefore were interested in determining the photomorphogenic growth of *cop1 cam7* double mutant. As a first step, we performed genetic crosses between *cam7* and *cop1‐4* or *cop1‐6* single mutants and generated *cam7*,* cop1‐4*, and *cam7*,* cop1‐6* double mutants. We then measured the hypocotyl length of 6‐day‐old seedlings grown in darkness or at various fluences of WL. The double mutants showed phenotype similar to *cop1‐4* or *cop1‐6* alleles in darkness as well as at various fluences of WL (Figure S2). These results suggest that additional loss of function of *CAM7* in *cop1* mutant background does not affect hypocotyl growth of *cop1* mutants.

### 3.2 Genetic interactions between *CAM7* and *COP1* modulate the physiological responses and light inducible gene expression

**Figure 2:**
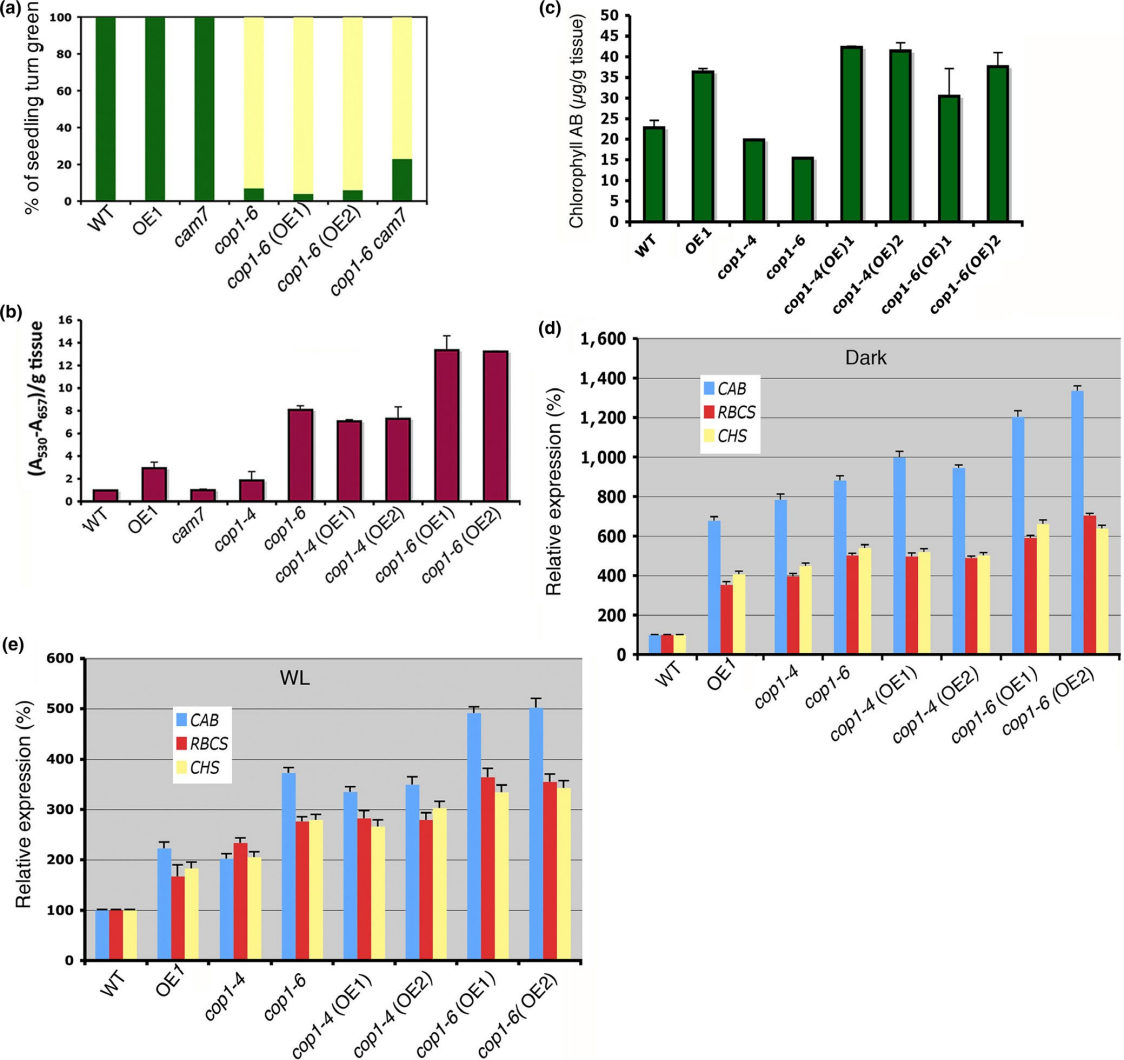
Overexpression of CAM7 in *cop1‐6* modulates physiological responses and gene expression of *cop1‐6*. (a) Quantification of percentage of seedlings turned green. Seedlings were grown for 5 days in the dark and transferred to WL (30 μmol m^−2 ^s^−1^) for 2 days. (b) Quantification of anthocyanin accumulation in 6‐day‐old seedlings grown in constant WL (60 μmol m^−2 ^s^−1^). (c) Accumulation of chlorophyll of wild‐type, mutant, and transgenic seedlings grown in white light (30 μmol m^−2 ^s^−1^). The error bars indicate *SD*. The number of independent experiments with similar results is (*n* ≥ 4). (d, e) Real‐time PCR analyses of *CAB1, RBCS1‐A, and CHS* transcript levels, from 6‐day‐old constant dark and white light (WL) grown seedlings, respectively. The data are first normalized with *ACTIN* and then presented against wild type as 100%. The error bars indicate *SD* (Student's *t* test, *<0.05). Number of independent experiments with similar results is (*n* ≥ 4)

Blocking of greening of seedlings is an important physiological parameter controlled by COP1 during the transition from dark to light conditions. While dark‐grown *cop1* mutant seedlings are transferred to WL, most of the seedlings are unable to turn green (Ang & Deng, 1994; Bhatia, Gangappa, Kushwaha, Kundu, & Chattopadhyay, 2008), whereas the wild‐type seedlings are able to turn green upon transfer to light from darkness. This blocking of greening phenotype of *cop1* mutant is allele specific, and becomes more prominent with longer the seedlings are grown in the dark (Ang & Deng, 1994; Holm et al., 2002). To determine the effect of *CAM7* mutation and its overexpression on the *cop1*‐mediated blocking of greening phenotype, we examined the blocking of greening effect in *cop1‐4 cam7, cop1‐4 CAM7OE*,* cop1‐6 cam7*, and *cop1‐6 CAM7OE* backgrounds. While 5‐day‐old dark‐grown seedlings were transferred to light for 2 days, *cop1‐6* and *cam7* mutant seedlings showed about 7% and 100% green phenotype, respectively; whereas *cop1‐6 cam7* double mutants exhibited ~20% of green seedlings (Figure 2a). These results suggest that *cam7* partly suppresses the blocking of the greening phenotype of *cop1‐6* mutant*s*. No such effect on *cop1*‐mediated blocking of greening was observed in *cop1‐4 cam7* double mutant seedlings (Figure S3).

Earlier studies have shown that similar to *cop1* mutants CAM7OE lines accumulate higher level of anthocyanin compared to wild‐type background (Kushwaha et al., 2008). Therefore, we wanted to test whether overexpression of CAM7 in *cop1* mutant background can modulate the anthocyanin level of *cop1* mutant. The examination of anthocyanin accumulation revealed that *cop1‐4 CAM7OE* and *cop1‐6 CAM7OE* transgenic lines had higher levels of anthocyanin than *CAM7OE*,* cop1‐4* and *cop1‐6* backgrounds (Figure 2b), suggesting that higher level of CAM7 in *cop1* mutant background enhances the anthocyanin accumulation of *cop1*.

Chlorophyll accumulation in *cop1* mutant background is reported to be decreased compared to wild‐type (Deng & Quail, 1999). To determine the chlorophyll accumulation in *cop1* transgenic lines overexpressing CAM7*,* we measured the total chlorophyll contents in 6‐day‐old seedlings grown in WL. The overexpression of CAM7 in *cop1* mutants caused a significant increase in chlorophyll content compared to *CAM7OE* and *cop1* mutant backgrounds (Figure 2c). To determine the genetic relationship between *CAM7* and *COP1* in the regulation of light inducible gene expression, we examined transcript levels of several light‐inducible genes: *CAB1, RBCS‐1A*, and *CHS1* in constant dark or WL conditions by quantitative real‐time PCR. For this, we used 6‐day‐old seedlings grown in the dark or WL conditions. In constant dark or WL, the expression of *CAB1, RBCS‐1A*, and *CHS1* was increased in *CAM7OE*,* cop1‐4*, and *cop1‐6* mutant lines as compared to the wild‐type background. The level of expression of these genes was further increased in *cop1‐4 CAM7OE* and *cop1‐6 CAM7OE* transgenic lines in the dark or WL grown seedlings (Figure 2d,e). Taken together, these results suggest that higher level of CAM7 additively enhances the expression of light inducible genes in *cop1‐4* and *cop1‐6* backgrounds.

### 3.3 Light regulates the nucleo‐cytoplasmic partitioning of CAM7 in arabidopsis hypocotyl cells

**Figure 3:**
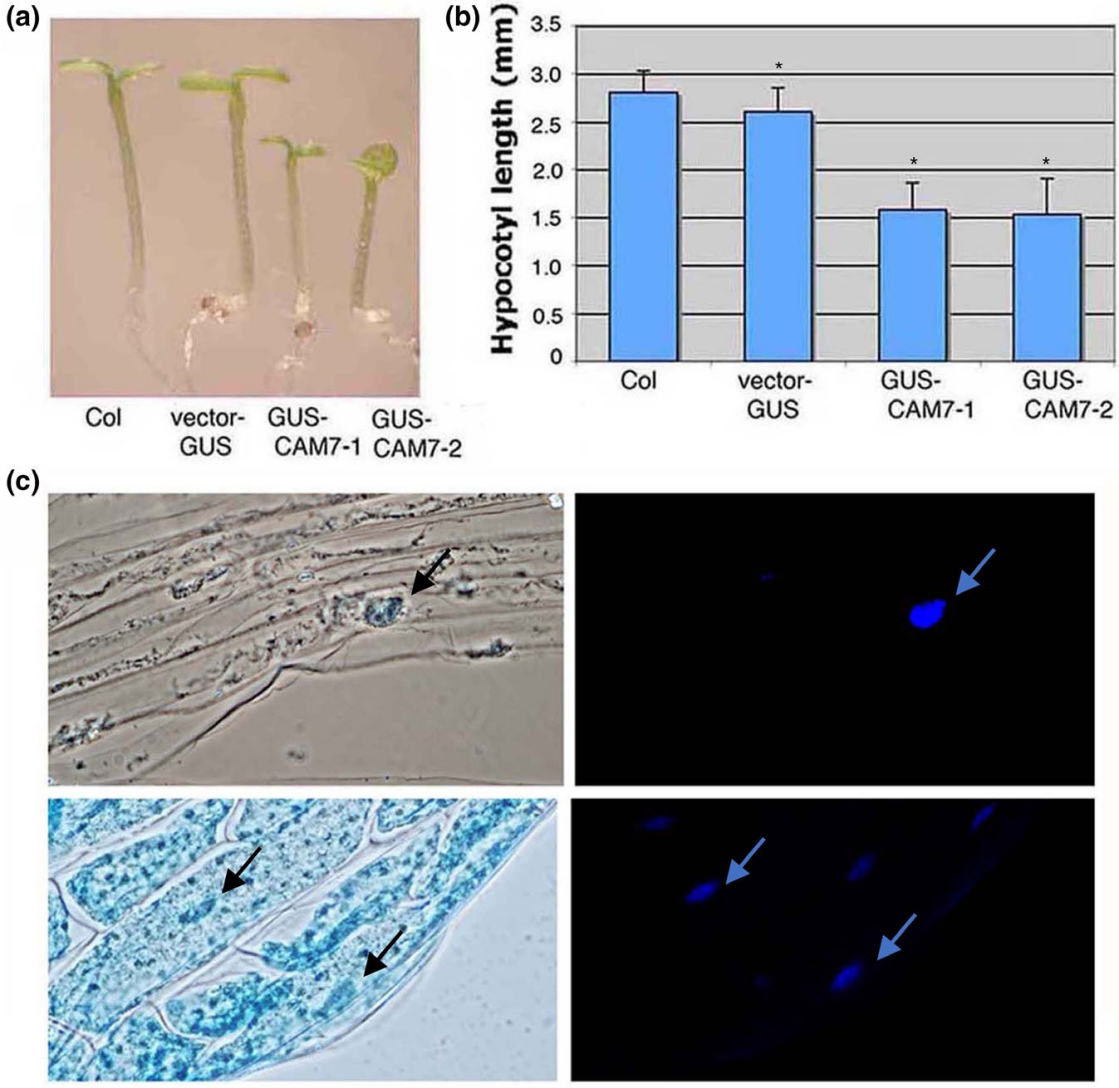
Light intensity‐controlled sub‐cellular localization of CAM7 in the hypocotyl cells of *Arabidopsis* transgenic seedlings. (a) Visible phenotype of 6‐day‐old wild‐type, transgenic lines containing *GUS*‐transgene alone (GUS), or two independent transgenic lines containing *GUS‐CAM7* transgene (*CAM7‐GUS (1) and CAM7‐GUS (2)*) grown in constant WL (30 μmol m^−2 ^s^−1^). (b) Quantification of hypocotyl length of seedlings shown in the right panel. The error bars indicate *SD* (Student's *t* test, *<0.05). The number of independent experiments with similar results is (*n* ≥ 3). (c) In each panel, the hypocotyl of transgenic seedlings were stained for GUS (left panels), and for DNA using DAPI to identify nuclei (right panels). The upper panels indicate hypocotyl cells of 6‐day‐old dark‐grown seedlings containing *GUS‐CAM7* transgene, and the lower panels indicate hypocotyl cells of 6‐day‐old WL (30 μmol m^−2^ s^−1^) grown seedlings. The arrows indicate the position of nuclei

COP1 is localized in the nucleus in darkness, and upon stimulation by light it translocates into the cytoplasm in a light‐intensity dependent manner (Huang, Yang, Ouyang, Chen, & Deng, 2014; Lau & Deng, 2012; Von Arnim & Deng, 1994). We were interested in investigating whether CAM7 subcellular localization is also controlled by light. To address this question, we generated transgenic lines containing either *GUS* alone or *GUS‐CAM7* regulated by *CaMV‐35S* promoter. To examine the functionality of GUS‐CAM7 fusion protein, we measured the hypocotyl length of 6‐day‐old WL grown transgenic seedlings. The measurement of hypocotyl length revealed that 6‐day‐old WL grown transgenic seedlings had significantly shorter hypocotyl than wild‐type (Figure 3a,b). To determine the GUS staining pattern, we used hypocotyl cells of 5‐day‐old transgenic seedlings grown in constant dark or WL. In dark‐grown seedlings, the GUS staining was detected exclusively in the nucleus in hypocotyl cells (Figure 3c). However, constant light‐grown seedlings showed GUS stain in the cytosol as well as in the nucleus (Figure 3c), similar to COP1 (Huang et al., 2014; Lau & Deng, 2012; Von Arnim & Deng, 1994). These results suggest that nucleo‐cytoplasmic partitioning of CAM7 is regulated by light.

### 3.4 CAM7 Physically Interacts with COP1

**Figure 4:**
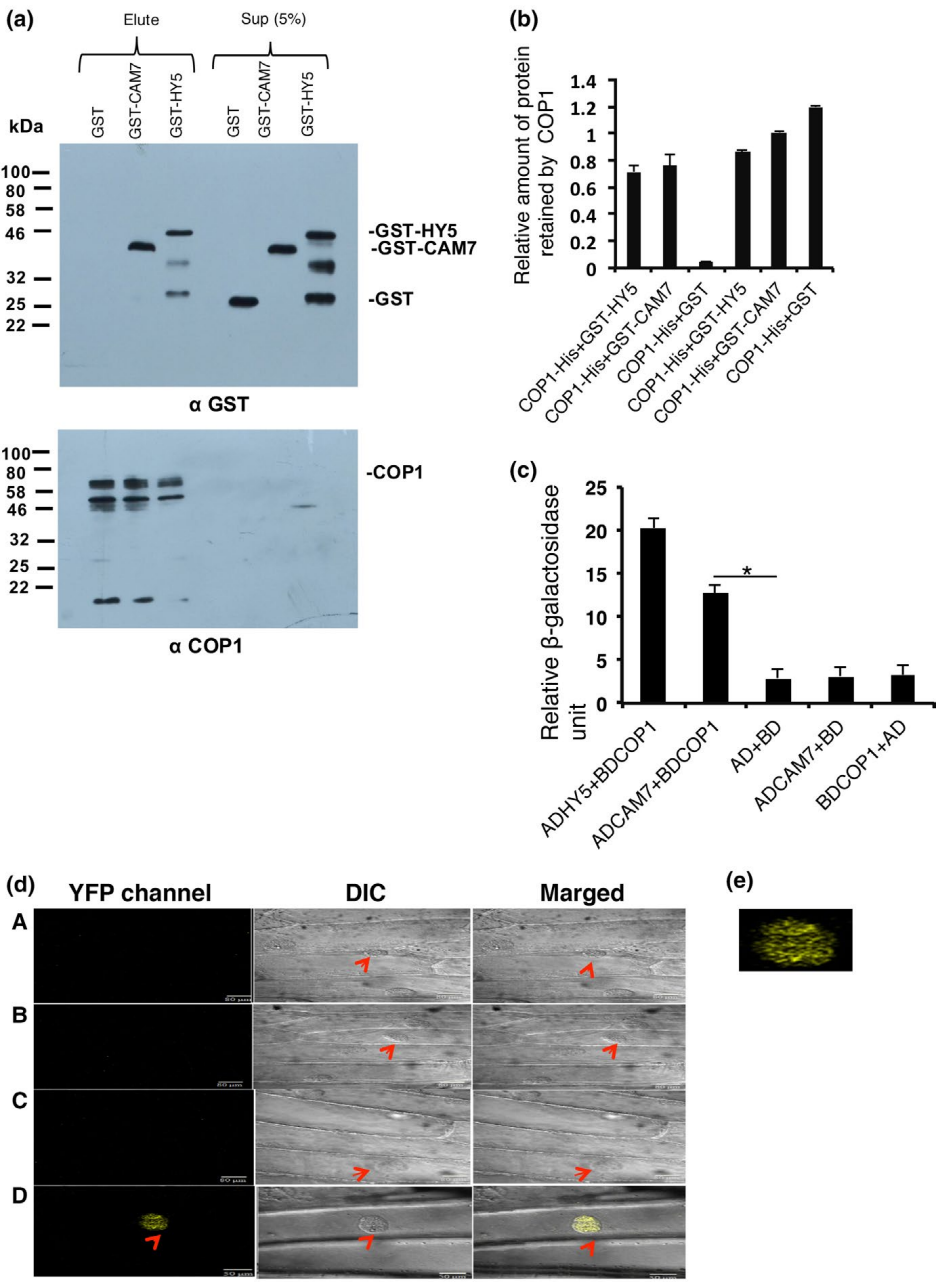
CAM7 Physically Interacts with COP1. (a) In vitro binding of CAM7 and COP1. GST‐HY5, GST‐CAM7, and GST proteins were individually incubated with Ni‐NTA bound 2 μg COP1‐6His protein in a fixed equimolar ratio. Supernatant and pellets were fractioned by 10% SDS–PAGE, blotted and probed with anti‐GST antibodies. Lane1 shows COP1‐6His with GST‐HY5 (positive control). Signal in supernatant serves as the loading control. The membranes were stripped and reprobed with polyclonal αCOP1 antibody (lower panel). (b) Quantification of protein retained by COP1‐His. The error bar shows the standard error of three independent experiments with similar results. (c) Yeast two‐hybrid interactions between CAM7 and COP1 was quantified by relative β‐galactosidase activity. Interaction between AD‐HY5 and BD‐COP1 used as positive control. The error bar shows the standard deviation of three technical replicates (Student's *t* test, *<0.05). Number of independent experiments with similar results is (*n* = 4). (d) BiFC assay for physical interaction between CAM7 and COP1. (A) Empty BiFC vector (B) CAM7‐cYFP and nYFP (C) COP1‐nYFP and cYFP (D) CAM7‐cYFP and COP1‐nYFP were cotransformed into onion epidermal cells. Left panel image shows the YFP channel image produced by reconstruction of YFP, middle panel shows the respective bright field image (DIC), Right panel image shows merged image. Arrow head indicates position of nuclei. (e) Zoom in picture of d (D), YFP channel

Since CAM7 and COP1 genetically interact, and both these proteins shuttle between the nucleus and cytosol in light dependent manner, we ask whether these two proteins physically interact with each other. Firstly, we performed in vitro‐binding assays using recombinant proteins. Approximately 2 μg of COP1‐His protein was individually incubated with the Ni‐NTA beads to examine the interactions with GST‐HY5 (used as positive control; Ang et al., 1998), GST‐CAM7 and GST alone. After incubation and washing, GST‐HY5, GST‐CAM7, and GST proteins were separately passed through columns containing Ni‐NTA beads attached to COP1‐His according to the respective molar ratios. The anti‐GST immunoblot showed that the amount of GST‐CAM7 retained by COP1‐His was comparable to GST‐HY5 and significantly higher than the background level retained by GST alone (Figure 4a,b). These results indicate direct physical interaction between COP1 and CAM7 in vitro.

To further substantiate the in vitro interaction, yeast two‐hybrid protein‐protein interaction assays were carried out. Full length COP1 was fused to the yeast GAL4 DNA‐binding domain (BD fusion), whereas the full length CAM7 was fused to the GAL4 activation domain (AD fusion). Fused DNA chimeric constructs with desired combinations were co‐transformed into AH‐109 yeast cells. Since physical interaction between COP1 and HY5 was demonstrated by two‐hybrid assay earlier (Ang et al., 1998), we used AD‐HY5 and BD‐COP1 as positive control in our study. The protein–protein interaction was quantified by relative β‐galactosidase assays using CPRG (chlorophenol red‐β‐d‐galactopyranoside) as a substrate as per Clonetech instructions. The chimeric AD‐fusion proteins, GAL4 activation domain with CAM7, HY5 (AD‐CAM7, AD‐HY5), were able to strongly activate the transcription of the *LacZ* reporter gene in the presence of the GAL4 DNA‐binding domain with COP1 (BD‐COP1; Figure 4c). However, no such activation was observed with other combinations of GAL4 activation and binding domain (Figure 4c).

To further test the observed physical interactions of CAM7 and COP1 in vivo, Bimolecular Fluorescence Complementation (BiFC) experiment was performed. For this, *CAM7* full‐length coding sequence was cloned to C‐terminal of YFP in pUC‐SPYCE vector, and *COP1* full length coding sequence to N‐terminal of YFP in pUC‐SPYNE vector. These constructs were co‐bombarded into the onion epidermal cells. The interaction between CAM7 and COP1 produced strong YFP florescence; whereas controls did not produce any YFP florescence (Figure 4d,e). The bright field image, and image merged with fluorescence confirm the position of nuclei. Taken together, these results suggest that CAM7 and COP1 physically interact in vivo.

### 3.5 Physical interaction of CAM7 and COP1 is light intensity dependent

**Figure 5:**
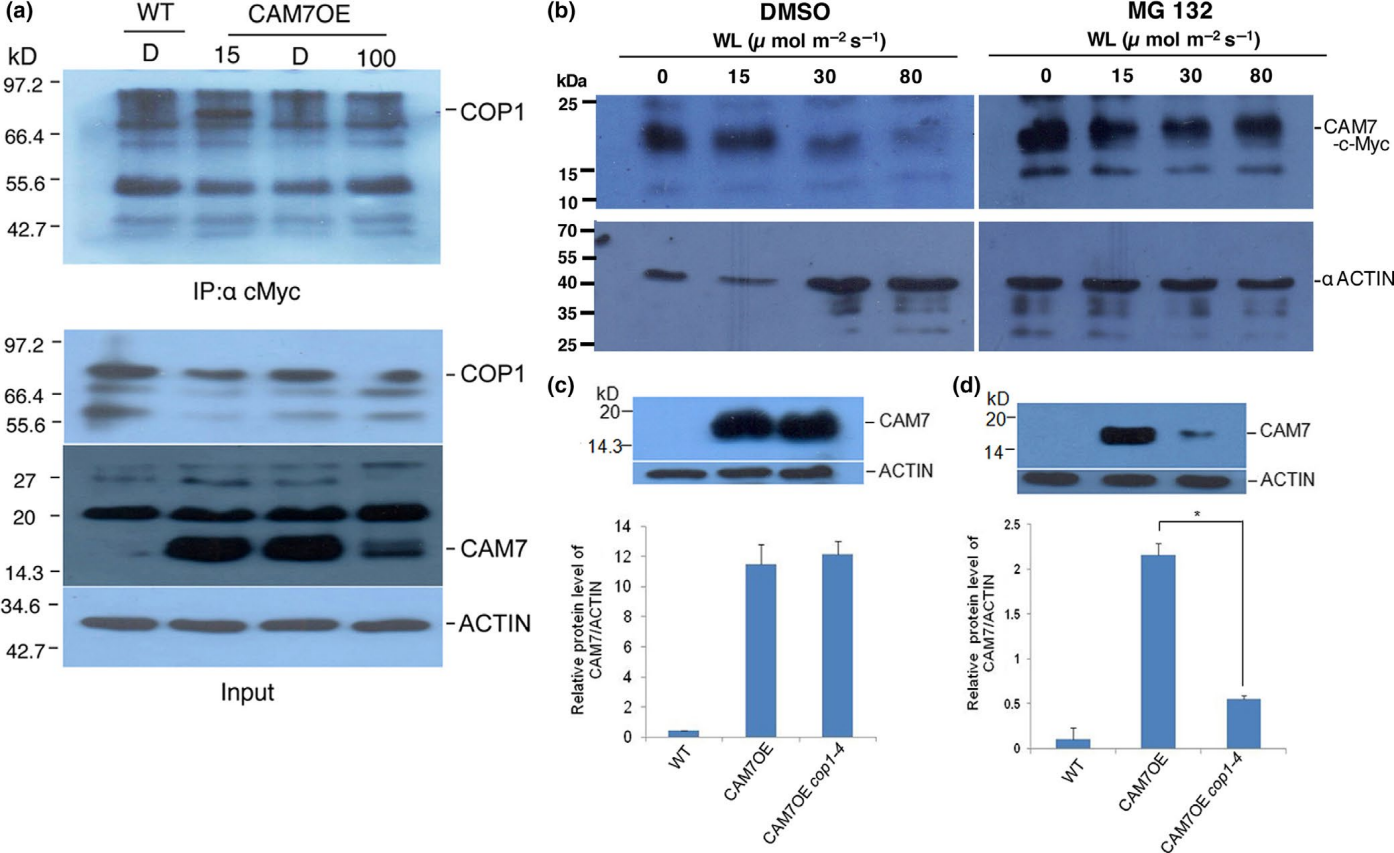
CAM7 stability in the dark is independent of COP1. (a) Immunoblot (using anti‐c‐Myc antibodies) of 500 μg of total protein prepared from wild‐type (WT) and CAM7OE seedlings grown in the dark (4‐day‐old white light‐grown seedlings transferred to the dark for 2 days (D)). The CAM7OE seedlings were grown at 15 μmol m^−2 ^s^−1^ (15) or 100 μmol m^−2 ^s^−1^ (100) of white light. The input controls are shown in the bottom panels. (b) CAM7 protein is stabilized by the 26S proteasome inhibitor (MG132). Five‐day‐old CAM7OE seedlings grown in the dark or constant WL conditions (15 to 80 μmol m^−2 ^s^−1^) were mock treated with 1% DMSO or treated with proteasomal inhibitor, MG132 for 12 hr. The seedlings were washed and the total protein was extracted and then subjected to immunoblot analysis using anti‐c‐Myc antibody. The location of CAM7‐c‐Myc is indicated. The membranes were stripped and reprobed with anti‐Actin antibodies. The lower panels show the immunoblot of anti‐Actin as loading controls. (c, d) Upper panels, Immunoblot (using anti‐c‐Myc antibodies) of 50 μg of total protein prepared from 6‐day‐old dark or WL grown seedlings (30 μmol m^−2 ^s^−1^), respectively, of wild‐type, *CAM7OE* and *CAM7OE cop1‐4*. Lower panels, Quantification of accumulation of CAM7 proteins shown in figures c and d, upper panels, respectively, relative to actin. Error bars represent *SE* (*n* = 3; Student's *t* test, *<0.01)

CAM7 protein accumulates at high level in dark‐grown seedlings, and upon exposure to WL, the accumulation of the protein gradually decreases (Abbas et al., 2014; Kushwaha et al., 2008). To determine whether the physical interaction between CAM7 and COP1 is light intensity dependent, we performed in vivo co‐immunoprecipitation (Co‐IP) assays using total plant protein extracts. The total plant protein extract was prepared from the wild‐type and CAM7‐cMyc overexpresser transgenic seedlings grown in dark‐adapted (4‐day‐old light‐grown seedlings transferred to the dark for 2 days) and constant WL conditions (15 and 100 μmol m^−2 ^s^−1^). CAM7 protein was immunoprecipitated using protein‐A agarose beads coupled to c‐Myc antibody. The analyses of the immunoblot using anti‐COP1 antibody showed that *CAM7OE* seedlings exhibited bands corresponding to COP1, grown at lower intensity, however, not in the dark or at higher fluences of WL (Figure 5a). A similar experiment was carried after seedlings were treated with proteasomal pathway inhibitor, MG132 to stabilize CAM7 at higher intensity of light. As shown in Figure S3, COP1 could be detected in seedlings grown at lower fluences, however, not in the dark or at higher fluences of WL. These results provide evidence that CAM7 interacts with COP1 in vivo at lower fluences of WL.

### 3.6 Functional COP1 is required for optimum CAM7 accumulation in WL

Since COP1 ubiquitin ligase physically interacts with CAM7, and the accumulation of CAM7 decreases at higher fluences of WL similar to COP1 (Kushwaha et al., 2008), we ask whether COP1 is involved in the regulation of CAM7 stability. To determine that, we first tested whether the lower level accumulation of CAM7 at higher fluences of WL was due to CAM7 degradation mediated by the 26S proteasome. We used proteasome inhibitor, MG132, for this study. As shown in Figure 5b, the level of CAM7 accumulation gradually decreased at higher fluences of WL in DMSO treated seedlings, consistent with the observation of Kushwaha et al., 2008;. On the other hand, higher level of CAM7 especially at 30 and 80 μmol m^−2 ^s^−1^ was accumulated after the treatment of MG132 under similar conditions (Figure 5b). These results suggest that CAM7 is degraded by the 26S proteasome‐pathway in WL.

We then tested the accumulation of CAM7 in *cop1‐4 CAM7OE* background in constant dark or WL conditions. Total protein was extracted from wild‐type, *CAM7OE* and *CAM7OE cop1‐4* seedlings, and Western blot analysis was performed using anti‐c‐Myc antibody. As shown in Figure 5c, no significant difference was observed in CAM7 level in *CAM7OE* and *CAM7OE cop1‐4* mutant backgrounds in the dark. However, in WL grown seedlings, the accumulation of CAM7 was reduced in *CAM7OE cop1‐4* background as compared to *CAM7OE* lines, indicating that functional COP1 is required for the optimum accumulation of CAM7 at lower intensity of WL (Figure 5d).

**Figure 6:**
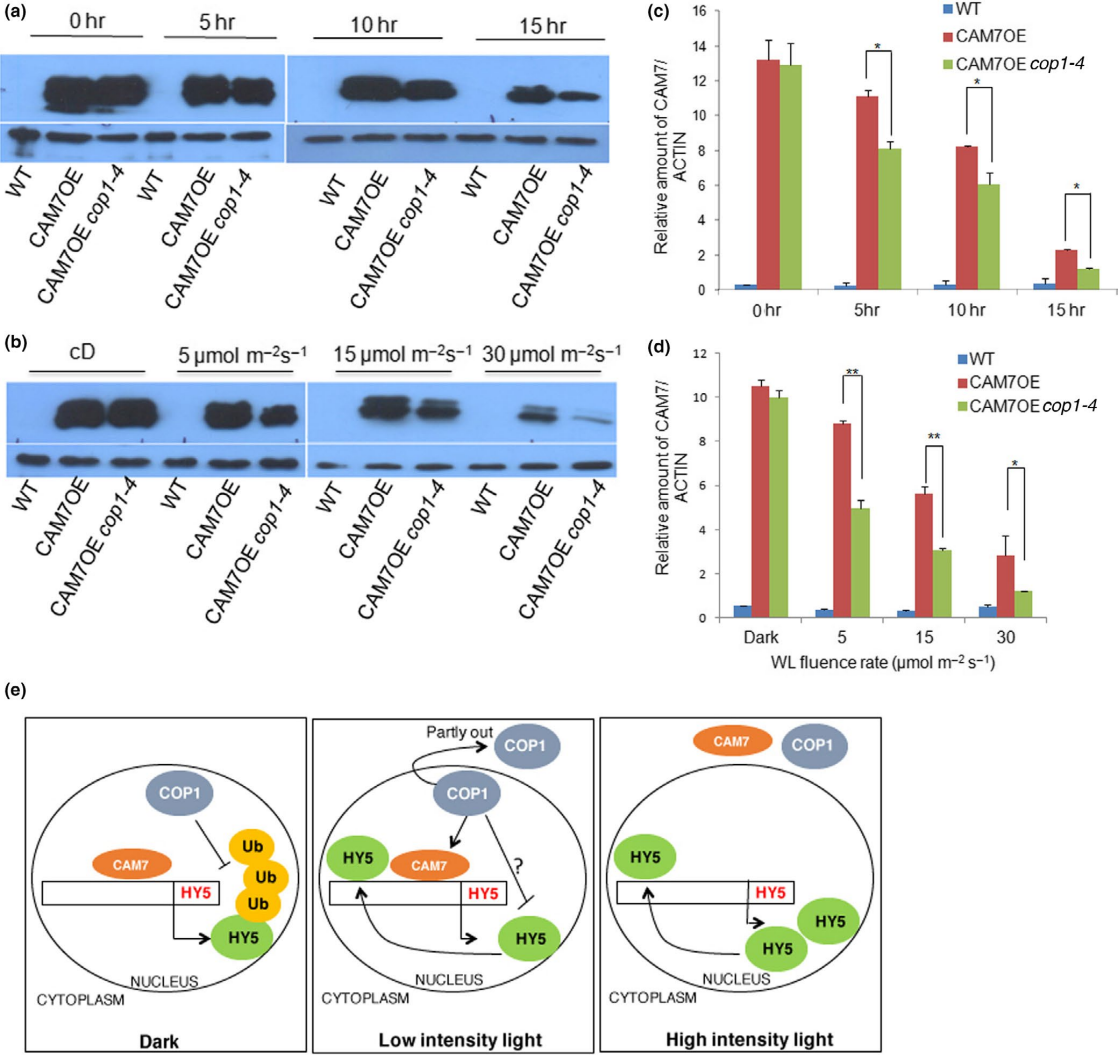
COP1 is required for the stability of CAM7. (a) Immunoblot to detect CAM7 protein isolated from 6‐day‐old dark‐grown WT,*CAM7OE*,*CAM7OE cop1‐4* seedlings transferred to WL (30 μmol m^−2 ^s^−1^) for 0, 5, 10, and 15 hr, respectively. (b) Immunoblot to detect CAM7 protein isolated from 6‐day‐old constant dark or 5, 15, and 30 μmol m^−2 ^s^−1^ of constant WL grown seedlings. Both the blots in a and b are probed with anti c‐Myc antibody. (c) Quantification of CAM7 protein levels after normalization with actin in wild type and various transgenic seedlings grown in the dark and transferred to WL (30 μmol m^−2 ^s^−1^) for different time points. (d) Quantification of CAM7 protein levels after normalization with actin in wild‐type and various transgenic seedlings grown in the dark and various intensities of WL. Error bars represent *SE* (*n* = 3; Student's *t* test, *<0.05; **˂0.01). (e) Proposed Working model. It has been shown earlier that HY5 and CAM7 bind to *HY5* promoter and promote the expression of *HY5* (Abbas et al., 2014). In the dark, COP1 degrades HY5 (Osterlund et al., 2000) while CAM7 being stable is involved in *HY5* expression. At lower fluences of WL, COP1 is partly out of the nucleus and the residual nuclear COP1 stabilizes CAM7. *HY5* is expressed at lower level and promotes photomorphogenesis weakly. At higher intensity of WL, COP1, and CAM7 are out of the nucleus, HY5 binds to its own promoter abundantly and is expressed at a high level to promote photomorphogenesis

To determine the kinetics of accumulation of CAM7 and compare its level between *CAM7OE* (in wild‐type background) versus *CAM7OE cop1‐4* backgrounds, we transferred 6‐day‐old dark‐grown seedlings to WL for various time points and monitored CAM7 level. As compared to *CAM7OE* background, CAM7 was detectable at lower levels in *cop1‐4 CAM7OE* seedlings, and the level of accumulation of the protein decreased with longer exposure to WL (Figure 6a,c). Importantly, the reduction in CAM7 level in *CAM7OE cop1‐4* lines was significantly higher than *CAM7OE* background (Figure 6a,c). To further test and expand our understanding about the stability of CAM7 protein in *CAM7OE cop1‐4* mutant background, we grew the seedlings in darkness or at various fluences of WL and performed immunoblot analyses. As shown in Figure 6b,d, CAM7 accumulation was gradually reduced with the increase in light intensity in *CAM7OE* background, and the level of reduction was further enhanced in *cop1‐4 CAM7OE* background. Taken together, these results demonstrate that functional COP1 is required for the stability of CAM7 at lower fluences of WL; however, its stability in the dark is independent of COP1.

## 4 DISCUSSION

It has been shown that HY5 and CAM7 together bind to T/G‐box and E‐box of *HY5* promoter, respectively, resulting in higher level of expression of *HY5* to promote photomorphogenesis (Abbas et al., 2014). It has been suggested from the previous studies that CAM7, which is abundant at lower fluences of WL, promotes *HY5* expression at lower fluences of WL (Abbas et al., 2014). The HY5 level is low at lower fluences of WL and once HY5 level increases with higher intensity of light, it binds to its own promoter to enhance its expression (Abbas et al., 2014; Osterlund et al., 2000). This study demonstrates that CAM7 and COP1, positive and negative regulators of photomorphogenesis, respectively, work in a coordinated manner to promote photomorphogenic growth. The concerted physiological functions of these two regulatory proteins were executed at the molecular level by physical interaction and stabilization of CAM7 by COP1.

The photomorphogenic growth phenotype of *cop1* mutants is light intensity dependent and allele specific (Ang & Deng, 1994). The higher level accumulation of CAM7 in *cop1* mutant (*cop1 CAM7OE*) enhanced the photomorphogenic growth as compared to *cop1* mutants in the dark and at lower fluences of various wavelengths of light. It is likely that a higher level of CAM7 in *CAM7OE* lines in dark increases the level of HY5, which is unable to be completely degraded by the endogenous COP1, and thus in turn HY5 promotes partial photomorphogenic growth as observed by Kushwaha et al. (2008) in darkness. In *cop1* CAM7OE background, this effect is further enhanced, and thereby the enhanced photomorphogenic growth is displayed. At lower fluences of light, while COP1 and CAM7 are still operative in the nucleus, CAM7 is stabilized by COP1, and this cooperative function between these two proteins enhances photomorphogenic growth and light regulated gene expression. It is worth mentioning here that earlier studies have suggested that COP1 can act as a positive regulator for the expression of light regulated genes at lower fluences of WL under certain circumstances (Chattopadhyay, Ang, Puente, Deng, & Wei, 1998). However, at higher fluences of light, both these proteins, COP1 and CAM7, are out of the nucleus (Figure 3; Von Arnim & Deng, 1994; Lau & Deng, 2012; Huang et al., 2014), and HY5, which is abundantly present, predominantly enhances its own expression to promote photomorphogenesis (Figure 6e).

The accumulation of CAM7 is light intensity dependent. It accumulates at higher level at lower intensity of light (Kushwaha et al., 2008). This study reveals that CAM7 is targeted by a proteasomal pathway (most effective at higher intensity of WL) since proteasomal pathway inhibitor such as MG132 was able to protect the degradation of the protein (Figure 5). It is likely that COP1‐mediated proteasomal pathway is not involved in the degradation of CAM7 since it rather stabilizes CAM7 at lower intensity of light, and moreover at higher intensity of light, COP1 is out of the nucleus (Huang et al., 2014; Lau & Deng, 2012; Von Arnim & Deng, 1994). Therefore, alternate proteasomal pathway components are likely to be operative to regulate the stability of CAM7 at higher intensity of WL.

Interestingly, earlier work showed that endogenous CaM was degraded by 26S proteasomes without ubiquitination (Tarcsa, Szymanska, Lecker, O'Connor, & Goldberg, 2000). One of the prerequisites for proteasomal degradation is the presence of an unstructured region in the substrate (Erales & Coffino, 2014; Jariel‐Encontre, Bossis, & Piechaczyk, 2008). Structural analysis and interaction studies of CaM demonstrated that it displays high conformational flexibility and itself undergoes from disorder to order transition (Barbato, Ikura, Kay, Pastor, & Bax, 1992; Kumar, Mazumder, Gupta, Chattopadhyay, & Gourinath, 2016; Radivojac et al., 2006; Tidow & Nissen, 2013; Wall, Clarage, & Phillips, 1997). In this case, the CaM may take up favorable geometry to facilitate interaction and translocation into the proteasome. Future study on CAM7 ubiquitination mechanism and interaction of CAM7 with 26S proteasome could provide further insight into the regulation of its turnover.

It was shown earlier that transcription factors of light signaling pathways such as PIF3 and GBF1 require COP1 for their stability in the dark and WL, respectively (Mallappa et al., 2008). Recently, COP1 has also been shown to prevent the proteasomal degradation of EIN3 by directly targeting EBF1 and EBF2 for ubiquitination (Shi et al., 2016). Linga, Lia, Zhua, and Deng (2017) have shown that COP1/SPA complex is associated with and stabilize PIF3 to repress photomorphogenesis in the dark. The higher level of CAM7 in *cop1* mutants results in hyper‐photomorphogenic growth (Figure 1). In a wild‐type scenario, COP1 stabilizes CAM7 at lower intensity of light (Figure 6), and thus works in a cooperative manner to promote photomorphogenesis at lower intensity of light (Figure 1). It is tempting to speculate that at lower intensity of light (where COP1 is still in the nucleus, at least partly), the level of HY5 is low and thus COP1‐mediated stabilized‐CAM7 interacts with the HY5 promoter to increase HY5 expression (Abbas et al., 2014). At higher intensity of light, COP1 is out of the nucleus (Huang et al., 2014; Lau & Deng, 2012) and CAM7 is degraded by a COP1‐independent proteasomal pathway (Figure 5). However, in this condition, the level of HY5 increases, and it more efficiently binds to its own promoter to be expressed at high level to promote photomorphogenesis (Figure 6e).

## AUTHOR CONTRIBUTIONS

D.S., R.K., J.P.M., and S.C. designed the research. D.S., R.K., J.P.M, S.D. S.B., and S.N.G. carried out the experiments. D.S., R.K., J.P.M., and S.C. analyzed the data and wrote the manuscript.
