# Supplementary material for: COP1 regulates the stability of CAM7 to promote photomorphogenic growth
Source: Plant Direct. 2019 Jun 25;3(6):e00144. doi: 10.1002/pld3.144 (PMC6593147; doi:10.1002/pld3.144)

**Supplemental Data**

**Figure S1.** Growth of *CAM7OE* in *cop1-4* or *cop1-6* transgenic seedlings at various wavelengths of light. **A**, Morphology of seedling grown in RL (15 μmol m^-2^ s^-1^), FR (20 μmol m^-2^ s^-1^), BL (15 μmol m^-2^ s^-1^). **B-D**, Quantification of hypocotyl length of 6-day-old seedlings grown in RL, FR and BL, respectively. Approximately 25 seedlings were taken for the measurement of hypocotyl length. The error bars indicate standard deviation (Student’s *t* test, * < 0.05). Number of independent experiments with similar results is (n≥3). **E,** Visible phenotypes of 6-day-old wild-type, mutants and transgenic seedlings grown in, RL (15 μmol m^-2^s^-1^), FR (20 μmol m^-2^s^-1^) and BL (15 μmol m^-2^ s^-1^). **F**-**G,** Quantification of hypocotyl length of 6-day-old seedlings grown in RL, FR and BL, respectively. Approximately 30 seedlings were used for the measurement of hypocotyl length. The error bars indicate SD (Student’s *t* test, * < 0.05). Number of independent experiments with similar results is (n≥3).

**Figure S2.** Growth of *cam7 cop1* double mutant seedlings in dark and at various wavelengths of light. **A**, qRT-PCR results show the level of expression of *CAM7* transcript in indicated backgrounds. The experiment was performed using cMyc (*CAM7cMyc_RP2*) and gene (*CAM7_iFP*) specific primers. Quantification analysis of the three biological replicates after normalizing to Actin is shown. Mean ± SD, n = 3. **B-C**, Morphology of 6-day-old seedling grown in dark or in WL (30 μmol m^-2^ s^-1^), respectively. **D-E**, Hypocotyl length measurement in dark and at various fluences of white light. Approximately 20 seedlings were used for the measurement of hypocotyl length. The error bars indicate SD (Student’s *t* test, * < 0.05). Number of independent experiments with similar results is (n≥3).

**Figure S3.** Physiological characterization of *cam7 cop1-4* double mutants and light dependent interaction between CAM7 and COP1. **A,** Visible block-of-green phenotype of indicated seedlings grown for 5-days in dark and transferred to WL (30 μmol m^-2^ s^-1^) for 2-days. **B,** Quantification of percentage of seedlings turned green. Error bars represent S.D. with no significance. The experiments were repeated for at least 2 times with consistent results and a representative result is shown. **C**, Immunoblot (using anti-c-Myc antibodies) of 500 μg of total protein prepared from wild type (WT) and CAM7OE seedlings grown in dark (4 days old white light grown seedlings transferred to dark for 2 days (D)). The *CAM7OE* seedlings were grown at 15 μmol m^-2^s^-1^ (15) or 100 μmol m^-2^s^-1^ (100) of white light. The input controls are shown in the bottom panels. CAM7 protein was stabilized by treating the seedling with proteasomal inhibitor MG132 for 12h. The seedlings were washed and total protein was extracted and then subjected for co-immunoprecipitation analysis. MW = molecular weights are in Kilo Dalton (kD).

**Figure S4.** Immunoblot to detect HY5 protein from 6-day-old dark adapted wild type (Col0) and *CAM7OE* seedlings. Four-day-old *CAM7OE* seedlings, grown in constant white light condition were dark adapted for 48 h. **A,** Total protein was extracted and subjected for immunoblot analysis using polyclonal anti-HY5 antibodies. The location of HY5 is indicated by asterisks. **B,** Ponceau staining of the Western blot is shown as loading control. **C,** Shows the immunoblot of anti-Actin as loading control. The membranes were stripped and re-probed with anti-Actin.

**Figure S1.** Growth of *CAM7OE* in *cop1-4* or *cop1-6* transgenic seedlings at various wavelengths of light. **A**, Morphology of seedling grown in RL (15 μmol m^-2^ s^-1^), FR (20 μmol m^-2^ s^-1^), BL (15 μmol m^-2^ s^-1^). **B-D**, Quantification of hypocotyl length of 6-day-old seedlings grown in RL, FR and BL, respectively. Approximately 25 seedlings were taken for the measurement of hypocotyl length. The error bars indicate standard deviation (Student’s *t* test, * < 0.05). Number of independent experiments with similar results is (n≥3). **E,** Visible phenotypes of 6-day-old wild-type, mutants and transgenic seedlings grown in, RL (15 μmol m^-2^s^-1^), FR (20 μmol m^-2^s^-1^) and BL (15 μmol m^-2^ s^-1^). **F**-**G,** Quantification of hypocotyl length of 6-day-old seedlings grown in RL, FR and BL, respectively. Approximately 30 seedlings were used for the measurement of hypocotyl length. The error bars indicate SD (Student’s *t* test, * < 0.05). Number of independent experiments with similar results is (n≥3).


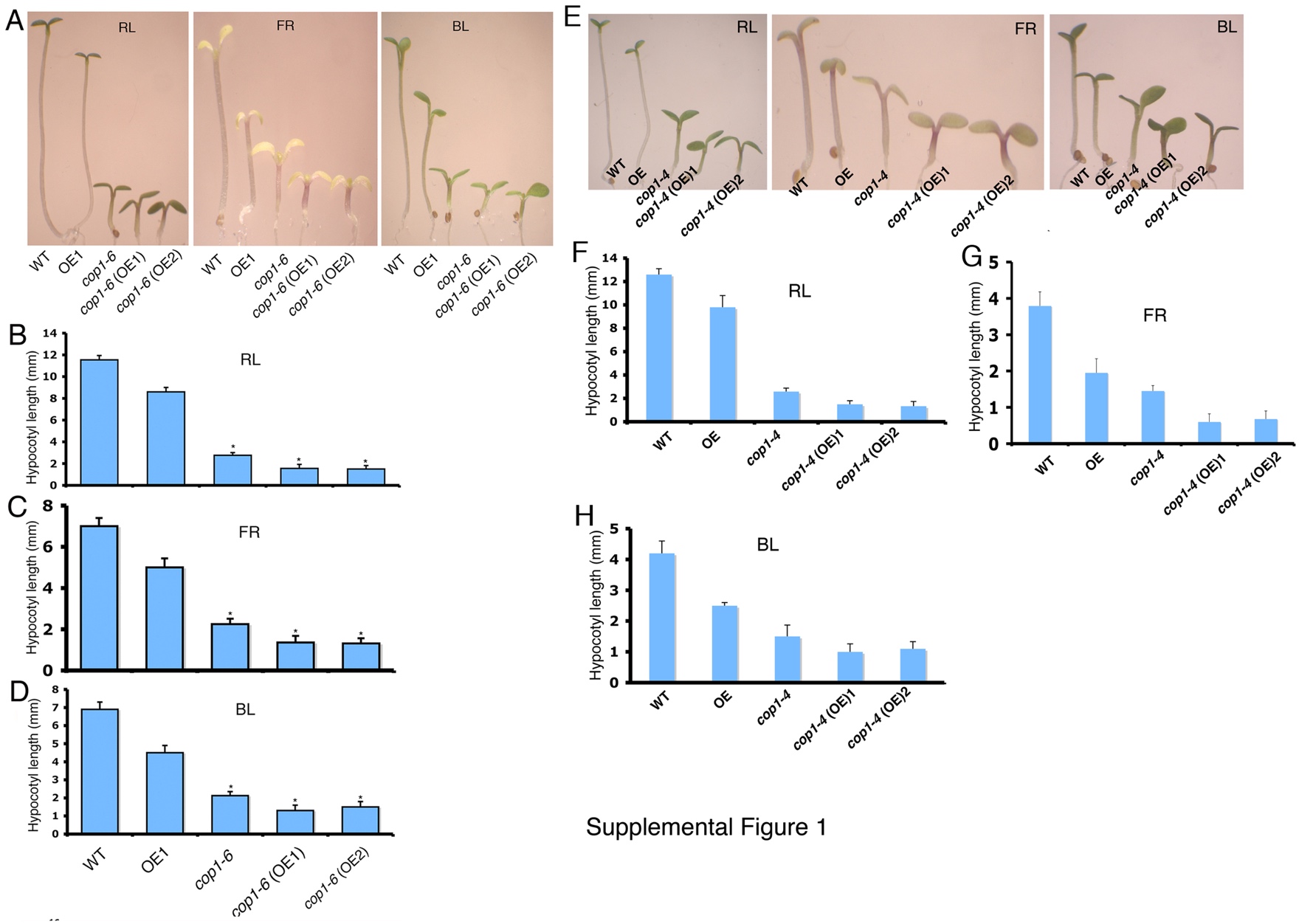


**Figure S2.** Growth of *cam7 cop1* double mutant seedlings in dark and at various wavelengths of light. **A**, qRT-PCR results show the level of expression of *CAM7* transcript in indicated backgrounds. The experiment was performed using cMyc (*CAM7cMyc_RP2*) and gene (*CAM7_iFP*) specific primers. Quantification analysis of the three biological replicates after normalizing to Actin is shown. Mean ± SD, n = 3. **B-C**, Morphology of 6-day-old seedling grown in dark or in WL (30 μmol m^-2^ s^-1^), respectively. **D-E**, Hypocotyl length measurement in dark and at various fluences of white light. Approximately 20 seedlings were used for the measurement of hypocotyl length. The error bars indicate SD (Student’s *t* test, * < 0.05). Number of independent experiments with similar results is (n≥3).


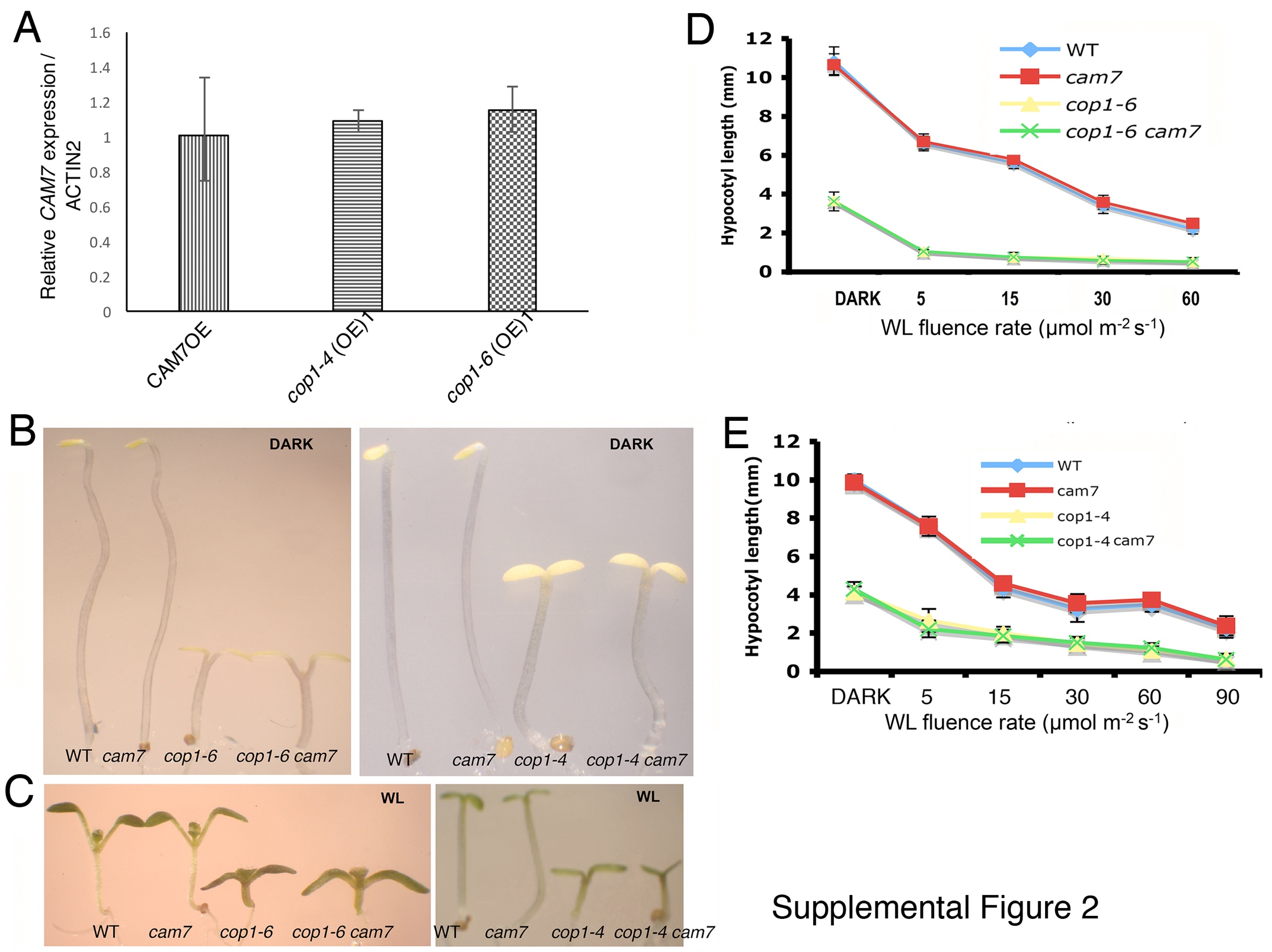


**Figure S3.** Physiological characterization of *cam7 cop1-4* double mutants and light dependent interaction between CAM7 and COP1. **A,** Visible block-of-green phenotype of indicated seedlings grown for 5-days in dark and transferred to WL (30 μmol m^-2^ s^-1^) for 2-days. **B,** Quantification of percentage of seedlings turned green. Error bars represent S.D. with no significance. The experiments were repeated for at least 2 times with consistent results and a representative result is shown. **C**, Immunoblot (using anti-c-Myc antibodies) of 500 μg of total protein prepared from wild type (WT) and *CAM7OE* seedlings grown in dark (4 days old white light grown seedlings transferred to dark for 2 days (D)). The *CAM7OE* seedlings were grown at 15 μmol m^-2^s^-1^ (15) or 100 μmol m^-2^s^-1^ (100) of white light. The input controls are shown in the bottom panels. CAM7 protein was stabilized by treating the seedling with proteasomal inhibitor MG132 for 12h. The seedlings were washed and total protein was extracted and then subjected for co-immunoprecipitation analysis. MW = molecular weights are in Kilo Dalton (kD).


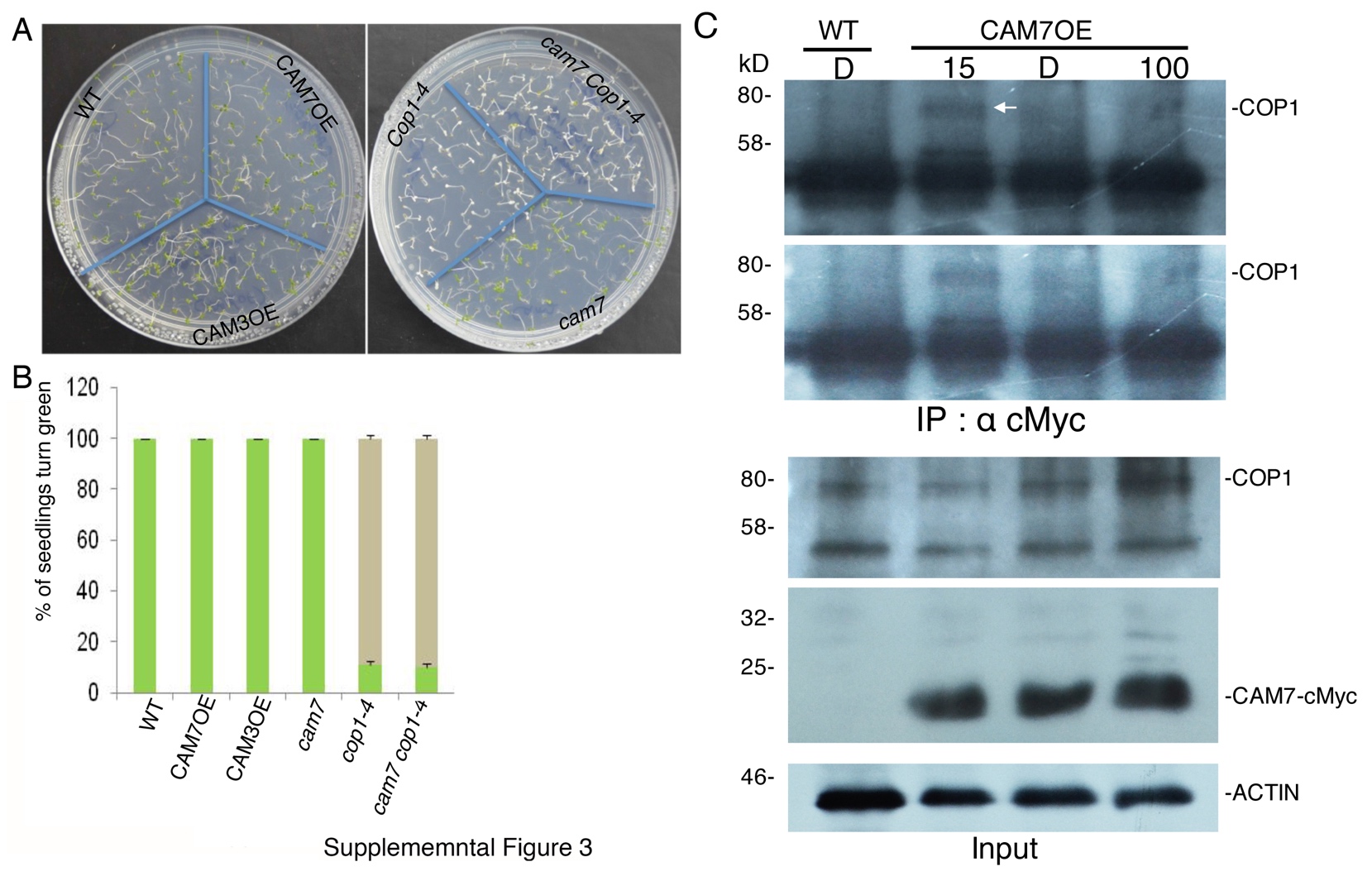


**Figure S4.** Immunoblot to detect HY5 protein from 6-day-old dark adapted wild-type and *CAM7OE* seedlings. Four-day-old *CAM7OE* seedlings, grown in constant white light condition were dark adapted for 48 h. **A,** Total protein was extracted and subjected for immunoblot analysis using polyclonal anti-HY5 antibodies. The location of HY5 is indicated by asterisks. **B,** Ponceau staining of the Western blot is shown as loading control. **C,** Shows the immunoblot of anti-Actin as loading control. The membranes were stripped and re-probed with anti-Actin.


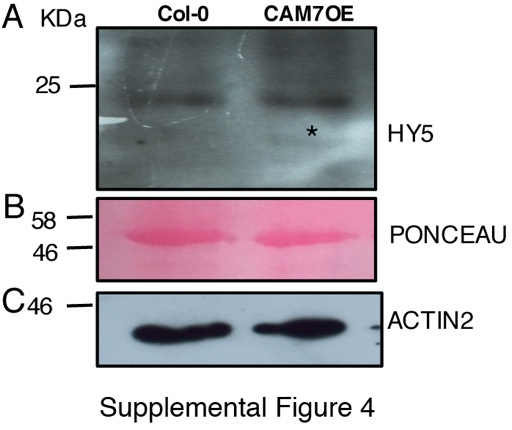

Supplement: Supplementary file 1 [file PLD3-3-e00144-s001.docx]
