# Supplementary material for: COP1 regulates the stability of CAM7 to promote photomorphogenic growth
Source: Plant Direct. 2019 Jun 25;3(6):e00144. doi: 10.1002/pld3.144 (PMC6593147; doi:10.1002/pld3.144)
Supplement: Supplementary file 2 [file PLD3-3-e00144-s002.docx]

Eva Farre

Hsoumin Li

Editor, Plant Direct

Dear Editors:

Thanks very much for your decision, and encouraging us to resubmit the revised version of the manuscript entitled “COP1 Regulates the Stability of CAM7 to Promote Photomorphogenic Growth” (Manuscript No.: Plant Direct: 201800228T). We thank you further for specifically summarizing the experimental requirements/modifications for the revised version of the manuscript. We have taken care of all the comments and suggestions of the reviewer. Please find below our point-by-point responses to the comments and suggestions of the reviewer. I hope that this revised version of the manuscript will be suitable for publication in Plant Direct.

Reviewer #1

In this manuscript Senapati et al., have demonstrated genetic and biochemical relationship between CAM7, a positive regulator of photomorphogenesis and COP1, which is a negative regulator of photomorphogenesis. They have demonstrated that CAM7 and COP1 interacts physically in light dependent manner, selectively in nucleus and that of overexpression of CAM7 in cop1 mutant background enhances photomorphogenic phenotypes of cop1 mutant. While the overall manuscript is well written and results presented here supports major conclusions of study, following modifications need to made to improve the quality of the manuscript.

**Major points**

1. Fig. 1 and part of Fig 2 are redundant. They can add Fig. 2F,G to Fig. 3 and delete Fig 2 as the hypocotyl data are presented in Fig 1 or move to supplementary if anything new is in this figure.

**Response**: We have moved Figure 2A-D to Supplementary Figure 1. This data is important since it shows the phenotypes at various wavelengths of light. We have moved Figure 2F and G to Fig 3 (revised MS Figure 2).

2. Seedlings images are of very poor quality, authors can present all the images against single background in which seedling phenotype can be visible clearly.

**Response**: We have replaced the images of dark grown seedlings of Figure 1 in the revised manuscript. The light grown seedlings look indeed very good in quality.

3. CAM7OE line has been crossed into cop14 and cop16 mutant background and used throughout the experiment. It is better to perform qRTPCR to make sure that transgenic CAM7 expression is similar amongst all background.

**Response**: We have included the data in the revised manuscript (Supplemental Figure 2).

4. In figure 3, qRTPCR is performed to analyze the expression of three genes under two different conditions and presented in two graphs. It might be better to prepare one graph each for a gene with both growth conditions into it. Moreover, authors mention relative expression in percentage (%), but neither method section nor figure legends clearly describe how it was normalized.

**Response**: We understand the point. However, since CAM7OE transgenic lines show partial photomorphogenesis in the dark, we examined the effect of all three gene-expression in dark and WL separately. We have stated the normalization procedure in figure legend of the revised manuscript.

5. In figure 4, the GUS images presented here are of low quality and lacks clarity.

**Response**: The original pictures are indeed well focused and of good quality. In the merged pdf it does not look that great, as rightly pointed out by the reviewer. Nonetheless, we have increased the pixels of the original pictures and also added arrows in the pictures to specify the results.

6. In figure 5A, in vitro binding of COP1HIS to GST tagged proteins, authors have shown only GST immunoblot image. Though authors might have used same source of COP1HIS for the experiment, HIS immunoblot or Coomassie blue staining of COP1HIS is necessary to convince readers that different lanes contain same amount of COP1HIS protein.

**Response**: We have performed the experiment again and included the data in revised manuscript, as suggested by the reviewer. In order to show that the lanes contain same amount of COP1 protein, we have probed by anti-COP1 antibodies. Please note that COP1 protein is known to be unstable and thereby we see multiple bands (Ang et al., 1998).

7. From figure 5D, it seems like BiFC signal show nuclear speckles, authors can zoomin on nucleus to determine if COP1CAM7 interaction in nucleus results in formation of nuclear speckles.

**Response**: We have added a close up picture of the data, as suggested.

8. In figure 6A CoIP experiment, level of CAM7myc in 100 umol m2s1 is significantly low. Authors can treat the seedlings with MG132 to stabilize the proteins first before doing CoIP. They can quantify from multiple blots and show average data. Moreover, figure legend doesn't match with figure. Authors need to correct the error. Also, they need to modify the abstract and result section to reflect that the light dependent interaction is in vivo data. In Fig. 5, they show COP1 interacts with CAM7 in a light independent manner. Thus, the light dependency is only in vivo. It is possible other factors are involved in this light dependent interaction.

**Response**: We have performed the experiment as suggested by the reviewer and the data has been included in Supplementary Figure 3. We have corrected the figure legend, and modified the statements as suggested.

9. HY5 immunblot in supplementary figure is unclear. Authors need to replace the blot image.

**Response**: We have replaced the Figure, however despite multiple attempts, we did not see a great improvement in visibility of HY5 protein. The result is although clear. The weakness may be in the HY5 antibody that we have used.

10. Most importantly, the manuscript lacks discussion about recent progress in the field. COP1 is shown to stabilize other proteins and also interact in a light dependent manner in vivo. Those literature should be discussed in this manuscript.

**Response**: We have included relevant recent references in the revised version of the manuscript.

Minor points

1. Fig. 5d, COPnYFP should COP1nYFP.

**Response**: We have corrected the mistake.

2. There are some spelling mistakes throughout the draft, please correct them.

**Response:** We have corrected all noticeable spelling mistakes in the revised manuscript.

3. Authors need to be consistent in terminology of wild type and transgenic plants throughout the draft.

**Response:** We have done so.

4. Transgenic plants name should be italicized.

**Response:** We have modified accordingly.
